# Neuroimaging of Sleep and Sleep Disorders

**DOI:** 10.3389/fneur.2014.00151

**Published:** 2014-09-29

**Authors:** Anne-Marie Landtblom

**Affiliations:** ^1^Department of Clinical and Experimental Medicine, Division of Neurology, County Council, University of Linköping, Linköping, Sweden; ^2^Department of Neuroscience, Uppsala University, Uppsala, Sweden

**Keywords:** imaging, narcolepsy, hypersomnia, neuronal circuitry, sleep disorders, physiology

Sleep physiology is a field of increasing importance because the recent awareness of how sleep affects us all, whether it is good or bad. Sleep disorders have a big impact on daily life and functioning, but this is true, also for other disorders that are not primarily associated with sleep disturbances. Subsequently, several disorders should be investigated and treated for sleep symptoms because these can be frequent. Examples here are Parkinson’s disease with frequent daytime sleepiness and insomnia. Also, epilepsy is frequently connected to disturbed night sleep in a complex way: antiepileptic drugs can be sedative, but seizures can also occur at night and disturb the night sleep. A focus on increased sleep health for persons with epilepsy can improve seizure control and should be included in future follow up of such patients. Of course, the impact of sleep deprivation on driving is a well-known problem that often concerns doctors, for example, in common diseases like obstructive sleep apnea syndrome, OSAS, as well as other chronic conditions, also regarding medications, with an impact on alertness. Naturally, sleep effects in healthy persons are of great importance in the society, concerning both safety and quality of life. Besides medicine, new fields also have emerged that do not primarily link to pathological conditions. The effects of sleep deprivation on economic decision making constitute an example of research that reveals how sleep can affect human behavior.

*Neuroimaging of Sleep and Sleep Disorders* is an outstanding book with high relevance to physicians and researchers interested in this area. It has a background part with introductory sections about *Neuroimaging of wakefulness and sleep*, *Neuroimaging*, *sleep loss and circadian misalignment*, and *Sleep and memory* containing the important imaging modalities, such as magnetic resonance imaging (MRI), positron emission tomography (PET), single-photon emission computed tomography (SPECT), sonography, magnetization transfer imaging (MTI), and combined EEG and functional magnetic resonance imaging (fMRI).

The core chapter thoroughly invents imaging experiences in a large variety of disorders and conditions connected to sleep disturbance: insomnia, depression, schizophrenia, narcolepsy (also including cataplexy as an isolated phenomenon), OSAS, parasomnia, central hypoventilation syndrome, fatal familial insomnia, posttraumatic stress disorder, sleepwalking, RLS, and sleep related epilepsies, as well as medication with sedating and alerting drugs.

Experts from the whole world contribute in this extensive book, edited by the well-known authorities Eric Nofzinger, Pierre Maquet, and Michael Thorpy. An impressive amount of researchers are represented in the author list: From Europe (Great Britain, the Netherlands, Italy, Sweden, Germany, Belgium, Spain, France, Austria, and Switzerland), from the USA (Andover, Charlestown, Philadelphia, Los Angeles, Pittsburgh, Albuquerque, Durham, Bronx, Boston), from Canada (Manitoba, Montreal, Winnipeg, Vancouver), from Asia (Singapore, Korea, Japan, China, India), and from Israel. In spite of the large amount of contributors and different subthemes, the text reads easily, and the content is well kept together through a good distribution of the material as well as excellent editing. This is a thorough inventory of fundamental and recent aspects of sleep medicine. Because of its great scope and span, this book is consequently recommended for all physicians who work in the field and for researchers. The information that is brought together in this book is highly comprehensive. There are examples of very recent contributions, so obviously the production process must have been speedy. In their preface, the editors point out that the book primarily was intended for sleep clinicians, radiologists, and researchers but that it is also suitable for neurologists, psychiatrists, for residents and fellows, graduate students, neuropsychologists, house officers, and even mental health and social workers who want to get a deeper understanding of this area. I would like to add general practitioners and pediatricians who often encounter these patients first. One third is devoted to methodology and data from normal individuals and two-thirds to data from disease states.

In his introduction, Eric Nofzinger sketches the situation of today: hypotheses regarding sleep medicine can now through development of imaging techniques be tested and further developed.

It is hard to highlight single contributions in this huge material, because it is all of great interest, but I would like to mention the chapter about sleep in schizophrenia and depression, which is of great interest and has the potential to make difference in the future clinical development.

Also, the field of neuroeconomy is nicely exemplified in an interesting article by Venkatraman and collaborators: *Sleep loss and circadian misalignment: Economic decision making in sleep deprivation*. See Figure 1.

**Figure 1:**
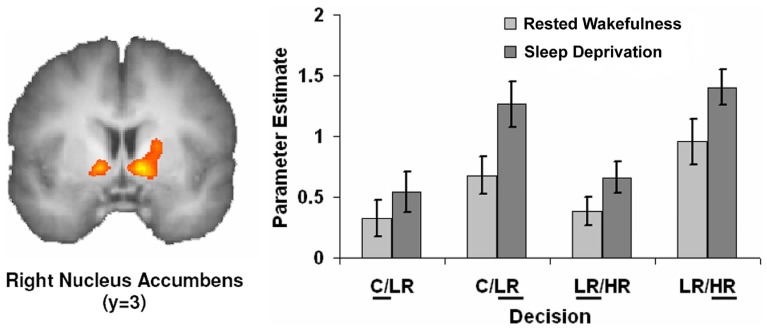
**Activation in nucleus accumbens tracks enhanced optimism following sleep deprivation**. Choosing the riskier option was associated with increased activation in the nucleus accumbens during the decision making phase in both states. There was a further significant increase in activation in this region following sleep deprivation for riskier choices, suggesting a greater expectation of being rewarded for these decisions when sleep deprived. Adapted from Vankatraman V, Chuah YM, Huettel SA, Chee MW. Sleep deprivation elevates expectation of gains and attenuates response to losses following risky decisions. *Sleep* (2007) 30(5):603–9 in Nofzinger E et al. Neuroimaging of sleep and sleep disorders, Figure 18.2. Reproduced with permission from the American Academy of Sleep Medicine and from Cambridge University Press.

This book will increase knowledge in all health practitioners that face patients with sleep disorders, and it is at the same time an appetizer for diving into different parts of this challenging field. This comprehensive edition is of great value for all professional sleep physicians and researchers in the area.

## Conflict of Interest Statement

The Guest Associate Editor Maria Engström declares that, despite having collaborated with the author Anne-Marie Landtblom, the review process was handled objectively and no conflict of interest exists. The author declares that the study was conducted in the absence of any commercial or financial relationships that could be construed as a potential conflict of interest.

